# Transient Retinal Vessel Whitening After Whole-Ventricle Radiotherapy

**DOI:** 10.7759/cureus.80178

**Published:** 2025-03-06

**Authors:** Takahiro Kogo, Yuki Muraoka, Hirohito Kubota, Masayuki Hata, Akitaka Tsujikawa

**Affiliations:** 1 Ophthalmology and Visual Sciences, Kyoto University, Kyoto, JPN; 2 Pediatrics, Kyoto University, Kyoto, JPN

**Keywords:** optical coherence tomography angiography, radiation retinopathy, retinal vessel whitening, ultra-widefield scanning laser ophthalmoscopy, whole-ventricle radiotherapy

## Abstract

We report a case of transient peripheral retinal vessel whitening following whole-ventricle radiotherapy in a pediatric patient, which resolved spontaneously without signs of ischemia. An 11-year-old girl with a pituitary germinoma underwent chemotherapy and whole-ventricle radiotherapy (23.4 Gy in 13 fractions). At two months post-treatment, ultra-widefield scanning laser ophthalmoscopy revealed bilateral peripheral retinal vessel whitening, raising suspicion of nonperfusion areas. However, optical coherence tomography angiography (OCTA) confirmed preserved retinal blood flow without ischemia. Visual field testing did not reveal any abnormalities. No intervention was performed, and the whitening resolved spontaneously within two months. Unlike typical radiation retinopathy, this represents a reversible, non-ischemic process, which could be attributed to the limited radiation exposure received during whole-ventricle radiotherapy and vascular plasticity in young patients. Given that radiation retinopathy is generally considered uncommon below 45 Gy, this case presents a characteristic retinal vascular change occurring at lower radiation doses and underscores the importance of retinal evaluation even in low-dose irradiation settings. Non-invasive imaging techniques, such as OCTA and ultra-widefield scanning laser ophthalmoscopy, can be valuable for monitoring subtle radiation-induced retinal changes. Further research is needed to refine screening and management strategies for radiation-induced retinal effects.

## Introduction

Radiation retinopathy is a chronic, progressive, occlusive vascular disorder primarily observed in patients who have undergone radiotherapy for head, neck, or intraocular tumors [1,2]. It arises from radiation-induced damage to the retinal vascular endothelium, resulting in increased vascular permeability, progressive capillary dropout, and eventual ischemia [3]. These circulatory disturbances manifest clinically as retinal hemorrhages, cotton-wool spots, neovascularization, and macular edema [1]. The onset of radiation retinopathy varies, typically occurring between 1 and 1.5 years after radiation exposure [2,4]. However, cases have been reported as early as three months post-radiation, particularly in patients receiving high total radiation doses, limited fractionation, or exhibiting increased individual susceptibility [4].

Retinal vessel whitening is often regarded as a marker of significant ischemia, indicating severe vascular damage [5]. In cases of extensive ischemia, irreversible interventions, such as laser photocoagulation, are frequently required to prevent disease progression and complications, such as neovascularization and vitreous hemorrhage [1].

Here, we present a unique case of retinal vessel whitening following radiotherapy for an intracranial lesion. Notably, despite the presence of retinal vessel whitening, there were no signs of ischemia, and the whitening resolved spontaneously over time without intervention. This suggests a distinct mechanism from conventional radiation retinopathy.

## Case presentation

**Figure 1:**
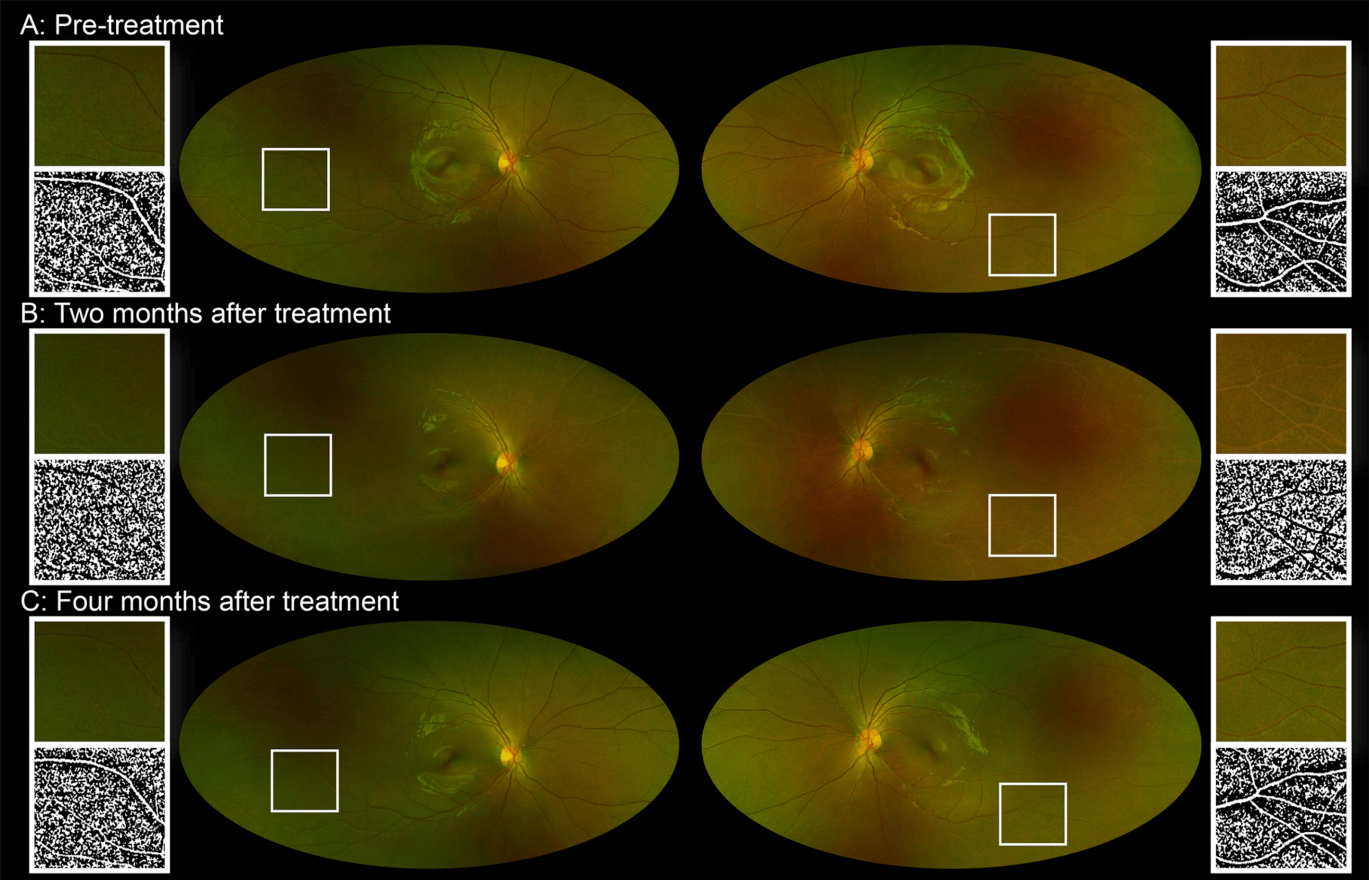
Changes in the retinal vasculature after whole-ventricle radiotherapy A. Ultra-widefield scanning laser ophthalmoscopy (SLO) images from the pre-treatment evaluation showing no abnormalities in the retinal vasculature. To enhance the visibility of the retinal vasculature in red, the green component was extracted using the ImageJ software ver. 1.51 (Wayne Rasband, National Institutes of Health, Bethesda, MD, USA), followed by inversion, noise reduction (Gaussian blur), and binarization (Niblack method). The processed images are also included. B. Ultra-widefield SLO images taken two months after whole-ventricle radiotherapy revealing bilateral pan-peripheral retinal vessel whitening that was not present prior to treatment. C. Ultra-widefield SLO images taken four months after whole-ventricle radiotherapy showing resolution of retinal vessel whitening and return of the retinal vasculature to its normal appearance.

An 11-year-old girl with pituitary germinoma underwent chemotherapy and whole-ventricle radiotherapy (23.4 Gy/13 fractions). Pretreatment ophthalmic examination revealed no abnormalities in the fundus or visual fields of either eye (Figure 1A).

**Figure 2:**
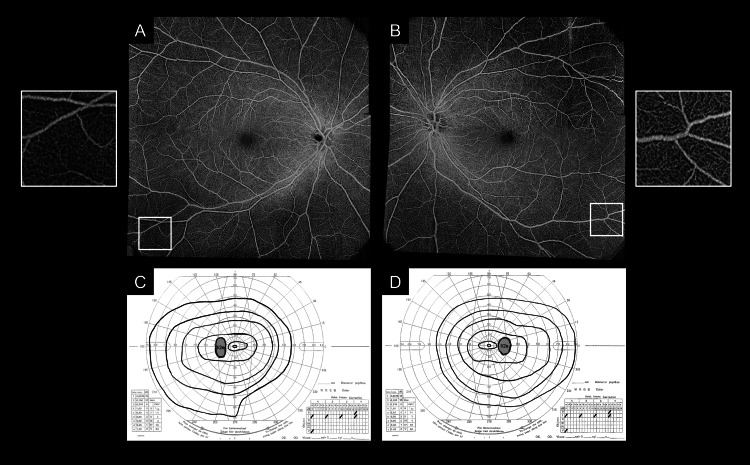
Optical coherence tomography angiography and visual field testing at two months at the time of retinal vessel whitening A, B. Optical coherence tomography angiography images with a scan area of 15 × 15 mm² showing preserved blood flow signals and no evidence of retinal nonperfusion areas, despite the presence of retinal vessel whitening. C, D. Visual field tests revealing no abnormalities.

Two months after radiotherapy, ultra-widefield scanning laser ophthalmoscopy (Optos California, Optos Plc, Dunfermline, UK) revealed bilateral, pan-peripheral retinal vessel whitening extending beyond the vascular arcades (Figure 1B), raising the suspicion of retinal nonperfusion areas (NPA). Fluorescein angiography was not performed because of concerns regarding a previous allergic reaction to the contrast agents used in computed tomography. Optical coherence tomography angiography (OCTA) (Plex Elite 9000, Carl Zeiss Meditec Inc., Dublin, USA) with a scan area of 15 × 15 mm^2^ showed preserved blood flow signals in the affected retinal vessels and confirmed the absence of NPA in both eyes (Figures 2A-2B). Visual field testing did not reveal any abnormalities (Figures 2C-2D). Blood examination at this time revealed a white blood cell count (WBC) of 1.79 × 10^9^/L, a red blood cell count (RBC) of 3.31 × 10^12^/L, and hemoglobin (Hb) levels of 10.0 g/dL. The C-reactive protein levels were <0.10 mg/dL, indicating mild anemia but no systemic inflammatory response. Additionally, there were no systemic inflammatory findings or clinical signs of systemic vasculitis. The patient was followed up without any intervention.

Two months later, all bilateral retinal vessel whitening resolved spontaneously, and OCTA confirmed no development of retinal NPA in either eye (Figure 1C). Blood examination demonstrated recovery of WBC to 4.07 × 10^9^/L, RBC to 3.59 × 10^12^/L, and Hb to 11.2 g/dL.

## Discussion

This case highlights a transient peripheral retinal vessel whitening without ischemia after whole-ventricle radiotherapy. Radiation retinopathy is typically a chronic and progressive occlusive vascular disorder associated with inflammation [3] and often requires treatments, such as corticosteroids, retinal photocoagulation, or anti-vascular endothelial growth factor agents, to address ischemic complications [1]. In this case, the retinal vessel whitening observed in the patient appeared to represent a milder form of vessel wall inflammation compared to typical radiation retinopathy, which is progressive and usually results in irreversible changes.

While radiation-induced vascular changes are well-documented, this case followed a self-limiting course, suggesting a different underlying mechanism. The patient exhibited a concurrent decline in RBC and Hb levels at the time of retinal changes, followed by spontaneous resolution of both anemia and vessel whitening as RBC and Hb levels recovered. This pattern suggests that systemic anemia may have influenced the visibility of the retinal vasculature, akin to conjunctival pallor observed in patients with anemia.

Given the known effects of radiation on endothelial integrity, we consider whole-ventricle radiotherapy-induced transient vascular inflammation as the most plausible explanation for these changes, with mild anemia playing a minor role in modulating vascular visibility. Additionally, as chemotherapy is known to increase the risk of radiation retinopathy [6], the possibility that chemotherapy may have exacerbated the effects of radiation therapy on the retinal vasculature should also be considered.

Although retinal vessel whitening is generally considered an irreversible finding, which is linked to retinal ischemia, it resolved spontaneously in this patient, suggesting a reversible pathology [5]. The peripheral localization and reversibility of the vessel whitening may reflect the localized effects of the radiation dose delivered during whole-ventricle radiotherapy and vascular plasticity in young patients. While frosted branch angiitis also involves reversible vessel wall changes [7], the findings in this case likely represent a distinct mechanism, highlighting the unique retinal effects of whole-ventricle radiotherapy in a young patient. This case emphasizes the potential for reversible retinal vessel whitening following radiotherapy, underlining the importance of careful observation and non-invasive imaging techniques, such as OCTA and ultra-widefield fundus imaging, for monitoring. The absence of ischemia and the spontaneous resolution of retinal changes suggest that not all cases of retinal vessel whitening necessitate immediate intervention.

Additionally, it is generally believed that radiation retinopathy does not develop at doses below 45 Gy, and fractionated irradiation reduces the risk of radiation retinopathy [2,6,8]. Hence, patients receiving lower doses of radiation are often not routinely examined for fundus changes. However, in this case, despite a relatively low dose of 23.4 Gy delivered in 13 fractions with whole-ventricle radiotherapy - a technique that further reduces retinal radiation exposure compared to whole-brain radiotherapy - retinal vascular changes still occurred. This suggests that previously unrecognized retinal changes due to radiation exposure may exist, highlighting the importance of retinal evaluation even at low radiation doses. As radiation retinopathy most commonly develops between 1 and 1.5 years after radiation therapy [2,4], regular follow-up examinations may be essential during this period. Wide-field fundus photography or slit-lamp examination can be useful for monitoring peripheral retinal vasculature, and if any changes are detected, further assessment with OCTA to evaluate blood flow may be warranted. Further research is essential to enhance the understanding of radiation retinopathy management and its long-term outcomes.

## Conclusions

This report describes a case of transient peripheral retinal vessel whitening after whole-ventricle radiotherapy in a pediatric patient, which resolved spontaneously without ischemia. Unlike typical radiation retinopathy, this case presents a reversible, non-ischemic process, which can be attributed to the limited radiation exposure received during whole-ventricle radiotherapy and vascular plasticity in young patients. These findings emphasize the importance of non-invasive imaging for monitoring and highlight the need for retinal evaluation even in patients receiving radiation therapy at lower doses. Further research is necessary to refine screening and management strategies for radiation-induced retinal changes.
